# Supplementary material for: Metabolic switch and epithelial–mesenchymal transition cooperate to regulate pluripotency
Source: EMBO J. 2020 Feb 24;39(8):e102961. doi: 10.15252/embj.2019102961 (PMC7156961; doi:10.15252/embj.2019102961)

Appendix

Metabolic switch and epithelial-mesenchymal transition cooperate to regulate pluripotency

Hao Sun[1,2,3,4,#], Xiao Yang[1,2,3,4,#], Lining Liang[1,2,3,4,#], Mengdan Zhang[1,2,3,4,#], Yuan Li[1,2,3], Jinlong Chen[1,2,3,4], Fuhui Wang[1,2,3,4], Tingting Yang[1,2,3,4], Fei Meng[1,2,3,4], Xiaowei Lai[1,2,3.4], Changpeng Li[1,2,3], Jingcai He[1,2,3], Meiai He[1,2,3,4], Qiaoran Xu[1,2,3,4], Qian Li[1], Lilong Lin[1,2,3.4], Duanqing Pei[1,2,3,4,5,*], Hui Zheng[1,2,3,4,5,*]

[*]Correspondence to: Hui Zheng and Duanqing Pei; Tel: 86-20-32015334; Fax: 86-20-32015231; Email: zheng_hui@gibh.ac.cn & pei_duanqing@gibh.ac.cn; #190 Kaiyuan Ave., Science City, Guangzhou, China, 510530.

[#]These authors contributed equally to this work

Table of Content

Appendix Figure S1-S10 with Legends

Appendix Figure S1

5C medium promotes the induction of pluripotency (related to Figure 1)

(A) The exogenous expression of four Yamanaka factors (*Oct4*, *Klf4*, *c-Myc*, and *Sox2*) were determined in 5C-Oct4GFP$^-$ cells and 5C-Oct4GFP$^+$ cells with qPCR.

(B) The protein levels of NANOG and REX1 were determined in two 5C-Oct4GFP$^+$ colonies with immunofluorescence. 5C-Oct4GFP- colonies have no detectable expression of these factors. Scale bar, 50 μM.

(C) The DNA methylation on the promoters of *Nanog* and *Oct4* were determined in 5C- GFP$^+$ cells with bisulfate sequencing.

Data information: Experiments were independently repeated at least five times (n≥5). Error bars represent standard deviations. Additional statistical information was listed in Dataset EV7.

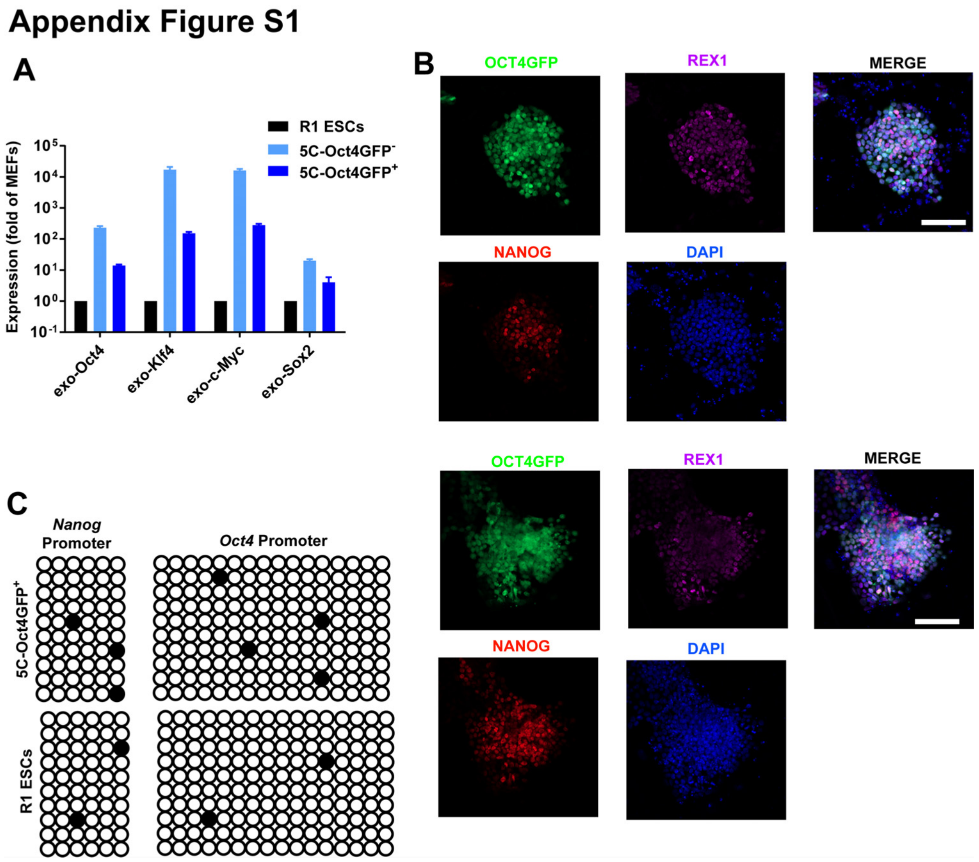

Appendix Figure S2

Energy metabolism is modulated by different methods (related to Figure 1)

(A) 5C or mES medium was used during reprogramming. On day 6, the expression of several glycolysis markers was determined with qPCR and normalized against those in MEFs.

(B) Reporter for HIF1α activity was delivered into MEFs 24 hours before reprogramming via a lentivirus system as described in Materials and Methods. The YFP fluorescence was determined at the beginning of reprogramming (MEFs or day 0) as control with FACS. The YFP fluorescence activity was also determined on day 3 and 6 during reprogramming with mES or 5C medium.

(C) The abilities of sh-RNAs (sh-Hif1α, sh-Pdk1, and sh-Pdk2) to suppress the expression of target genes were determined three days after being delivered into MEFs. The expression of target genes in cells with tested shRNAs were normalized against and compared with that in cells with sh-Luc.

(D-E) Expression of *Hif1α* was modulated with overexpression or sh-RNA-mediated knockdown via a retrovirus system. Energy metabolism was analyzed on day 6 during reprogramming with the Seahorse instrument. Increase in ECAR after adding glucose was considered as glycolysis ability of the cells, while decrease in OCR after adding oligomycin was considered as the ATP production ability of the cells.

(F-G) Energy metabolism was controlled by modulating the expression of *Pdk1/2* with a retrovirus system or using small-molecule compounds, oligomycin (1 μM) and 2-DG (5 mM). Energy metabolism was analyzed on day 6 during reprogramming with the Seahorse instrument. Glycolysis ability and ATP production ability of the cells were listed.

Data information: Experiments were independently repeated at least five times ($n \geq 5$) except Pscan analysis. Error bars represent standard deviations. ***$p < 0.001$. Additional statistical information was listed in Dataset EV7.

Appendix Figure S2

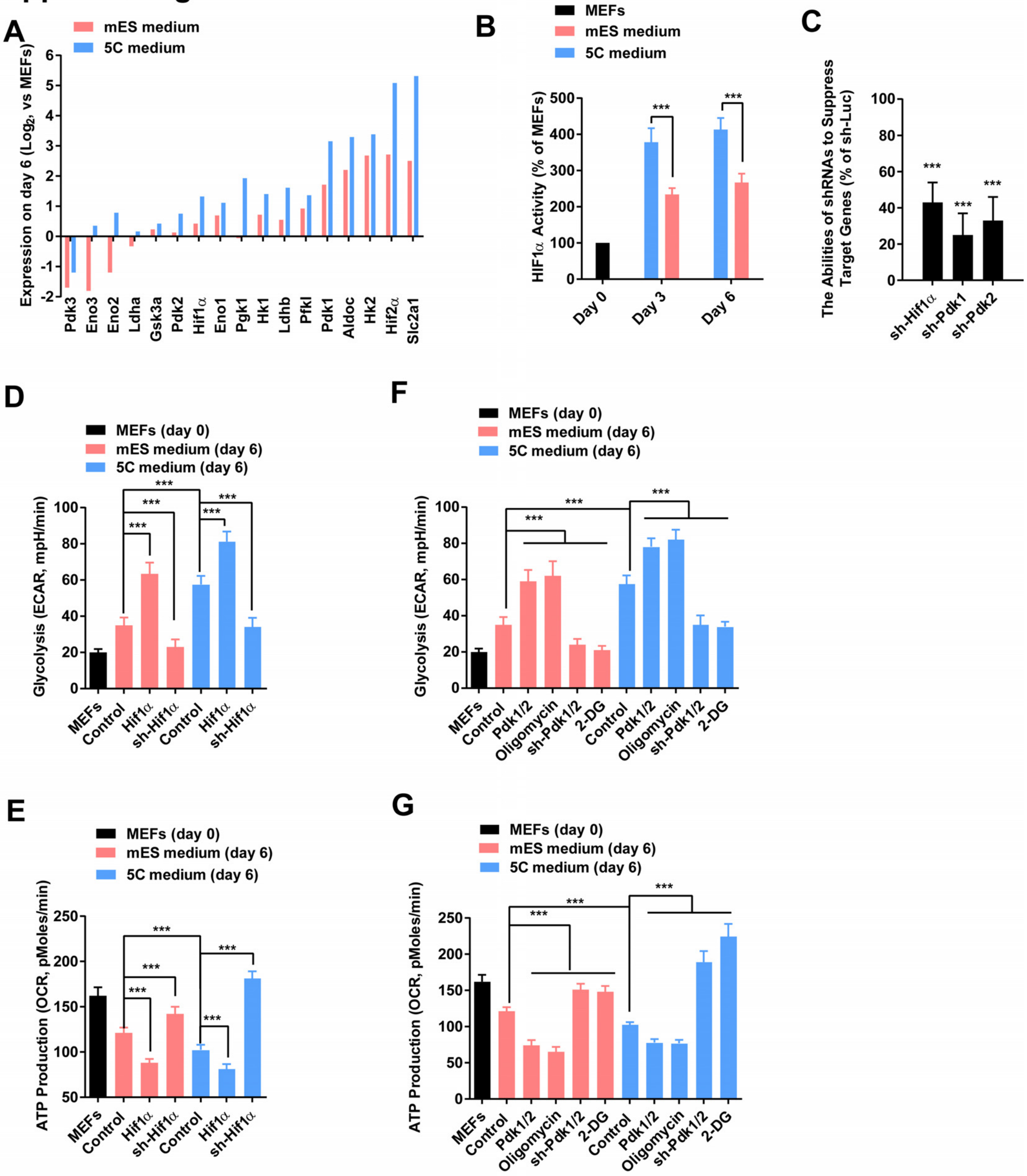

Appendix Figure S3

5C medium facilitates the reprogramming by reducing pre-iPSCs formation (related to Figure 2)

(A) The expression profiles of pre-iPSCs, iPSCs, and ESCs were obtained from previous reports (Data ref: Gene Expression Omnibus GSE10871, 2008; Data ref: Gene Expression Omnibus GSE14012, 2009). Genes with significant expression differences (Log_2 value change over 1.5) between pre-iPSCs and iPSCs/ESCs were selected (heatmap on left). The expression of these genes in pre-iPSCs, iPSCs, and ESCs were normalized against those in MEFs and plotted in heatmap in middle. Similarly, after normalizing against corresponding gene expression in MEFs, the expression of these genes in 5C-Oct4GFP$^-$ and mES-Oct4GFP$^-$ cells were listed and plotted (current RNA-Seq, heatmap on right).

(B) Four types of expression barriers during the conversion from pre-iPSCs to iPSCs were summarized. The genes which were required to be down- or up-regulated during reprogramming (from MEFs to iPSCs) but were not down- or up-regulated in pre-iPSCs, were considered as type I or II barriers. The genes which expressed at similar levels in MEFs and iPSCs but were significantly up- or down-regulated in pre-iPSCs, were considered as type III or IV barriers. The numbers of barriers that have already been overcome or not identified in 5C-Oct4GFP$^-$ and mES-Oct4GFP$^-$ were summarized.

(C) The expression of typical methyltransferases and demethylases targeting H3K9 were determined in 5C-Oct4GFP$^-$ and mES-Oct4GFP$^-$ cells with qPCR on day 6 during reprogramming.

(D) The abilities of sh-RNAs (sh-Bmi1, sh-Ctcf, sh-Ezh2, sh-Kdm2b and sh-Wdr5) to suppress the expression of target genes were determined on day 6 during reprogramming with 5C medium. The expression of target genes in cells with tested shRNAs were normalized against and compared with that in cells with sh-Luc.

(E) The abilities of sh-RNAs (sh-Snai2, sh-Twist1, sh-Twist2, and sh-Zeb1) to suppress the expression of target genes were determined three days after being delivered into MEFs. The expression of target genes

in cells with tested shRNAs were normalized against and compared with that in cells with sh-Luc.

Data information: Experiments were independently repeated at least five times (n≥5) except dataset analysis. Error bars represent standard deviations. *p < 0.05, **p < 0.01, ***p < 0.001. Additional statistical information was listed in Dataset EV7.

Appendix Figure S3

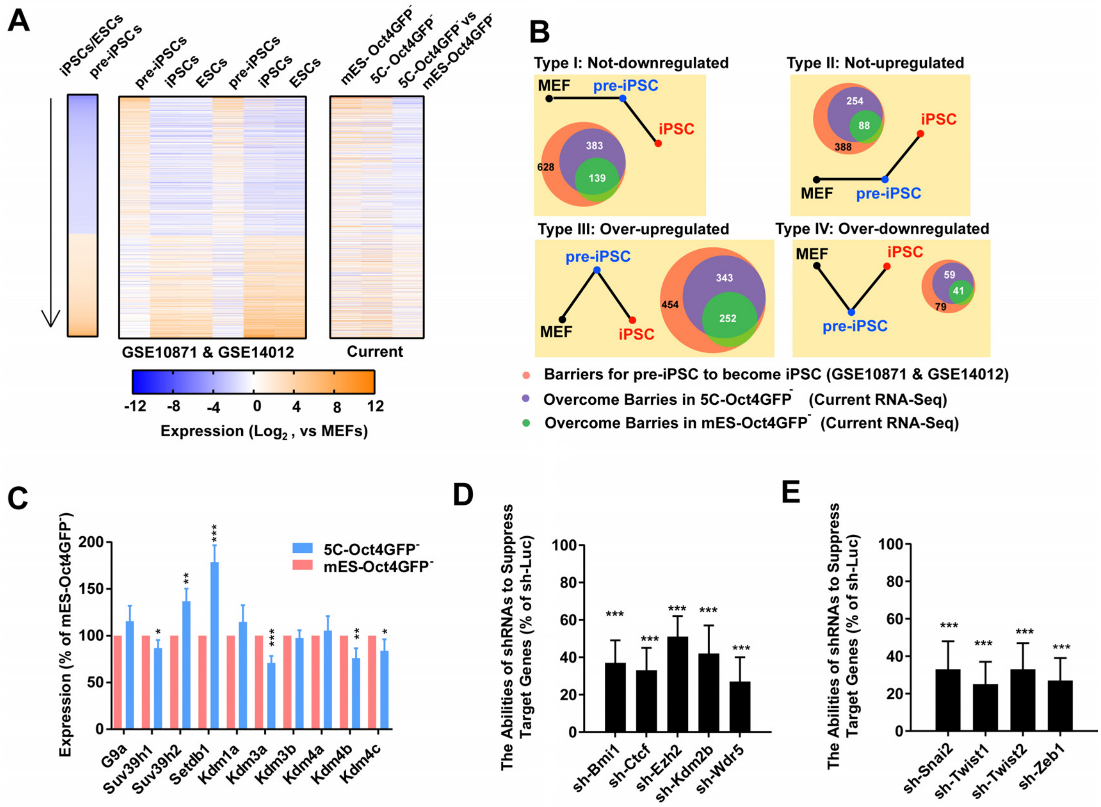

Appendix Figure S4

Early EMT and OGS during other cell fate conversions (related to Figure 4)

Previous RNA-Seq results were analyzed to determine whether similar cooperation existed between early EMT and facilitated OGS during other cell fate conversions, like trans-differentiation from MEFs to functional neurons (Data ref: Gene Expression Omnibus GSE68902, 2016) and differentiation from human ESCs to hepatocytes (Data Ref: Gene Expression Omnibus GSE70741, 2017). Expression of several mesenchymal and glycolysis markers were presented in (A-B) and summarized as average results in (C-D) after normalizing against the corresponding expression on day 0. The expression of *Bmi1*, *Ctcf*, *Ezh2*, *Kdm2b*, and *Wdr5* was summarized in (E-F).

Data information: Additional statistical information was listed in Dataset EV7.

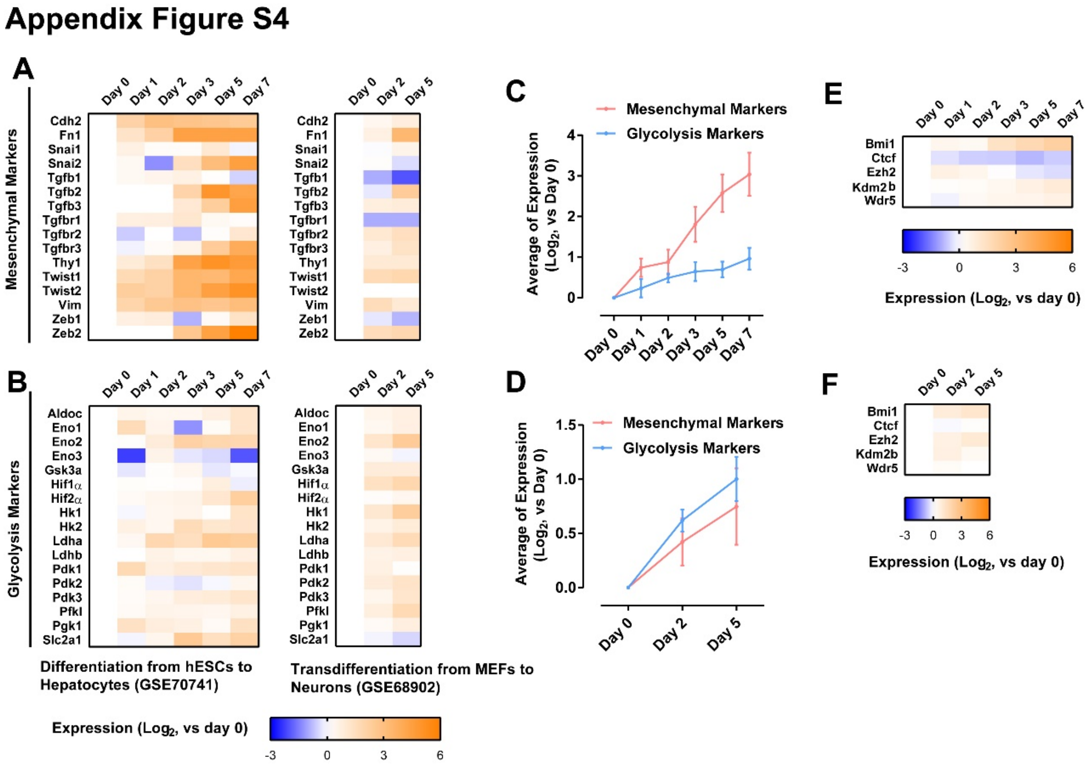

Appendix Figure S5

Metabolic state contributes to reprogramming directly (related to Figure 4)

(A-B) *Hif1α* was overexpressed via a retrovirus system during reprogramming with mES medium. Oligomycin (1 μM) was used in another group. The expression of *Bmi1*, *Ctcf*, *Ezh2*, *Kdm2b*, and *Wdr5* was determined on day 6 with qPCR (A). The expression of *Hif1α*, *Pdk1*, and *Pdk2* was determined on day 6 with qPCR (B).

(C) The expression of *Hif1α* was modulated with overexpression or sh-RNA-mediated knockdown via a retrovirus system. Energy metabolism was directly controlled by using small-molecule compounds, oligomycin (1 μM) and 2-DG (5 mM). These different treatments were combined during reprogramming with mES medium. The Oct4GFP$^+$ colonies were counted on day 15 and normalized against the number in group without any treatment.

(D-E) Early EMT and the expression of related transcriptional factors were regulated by using TGFβ (TGFβ1/2/3, 1ng/ml each) and Repsox (1 μM) on day 2-7. Experiments in (C) were repeated in the context of TGFβ (D) and Repsox treatment (E). The Oct4GFP$^+$ colonies were counted on day 15 and normalized against the number in group without any treatment (heatmaps on the left). Addition normalization against the corresponding results in (C) was performed and the final results were listed in heatmaps on the right to make the functions of TGFβ and Repsox clearer.

(F) The expression of *Bmi1*, *Ctcf*, *Ezh2*, *Kdm2b*, and *Wdr5* was modulated with overexpression or sh-RNA-mediated knockdown via a retrovirus system. Energy metabolism was directly controlled by using small-molecule compounds, oligomycin (1 μM) and 2-DG (5 mM). These different treatments were combined during reprogramming with mES medium. The Oct4GFP$^+$ colonies were counted on day 15 and normalized against the number in group without any treatment.

Data information: Experiments were independently repeated at least five times (n≥5). Error bars represent

standard deviations. *p < 0.05, **p < 0.01, ***p < 0.001. Additional statistical information was listed in Dataset EV7.

Appendix Figure S5

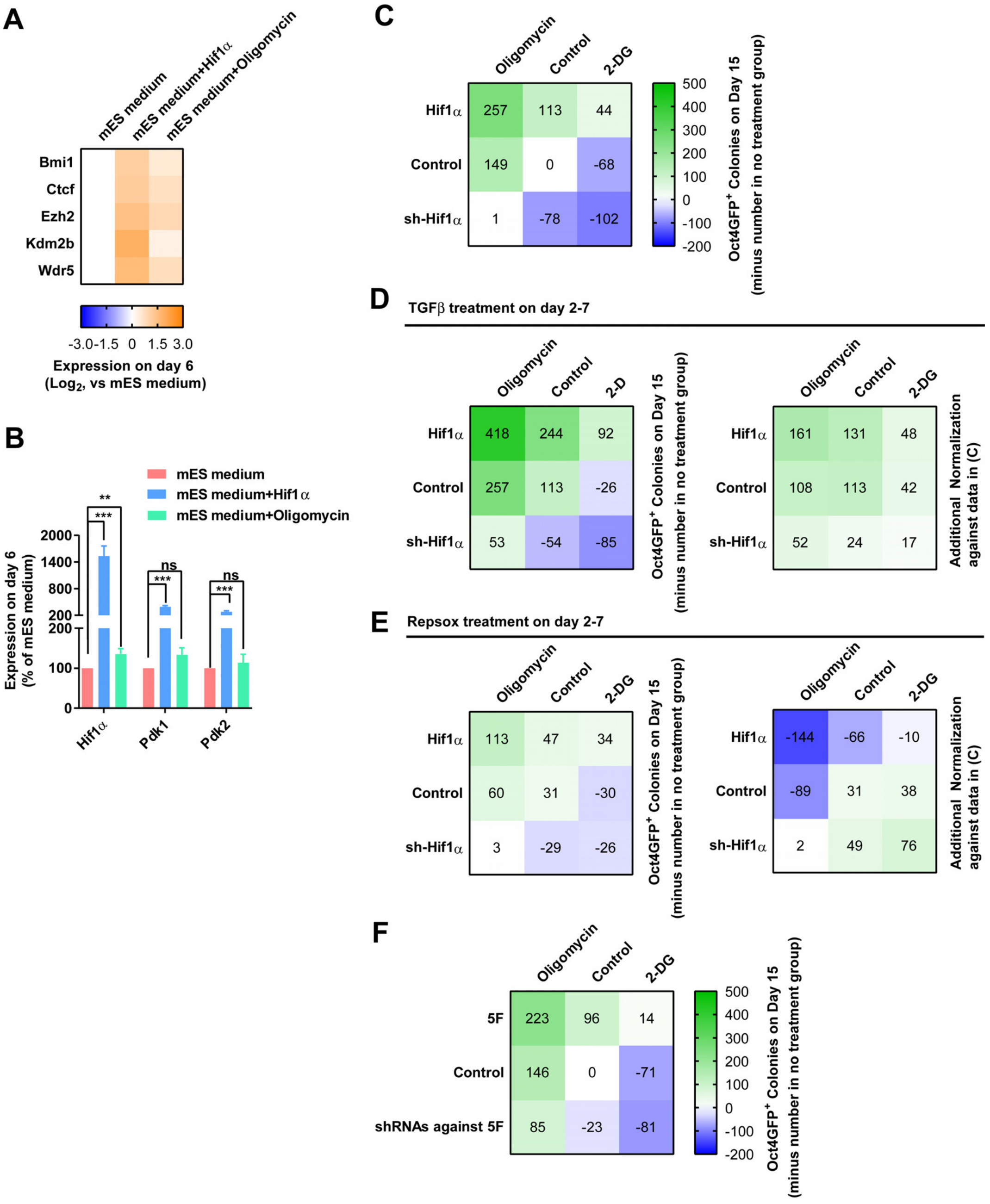

Appendix Figure S6

Early EMT and OGS are also observed during other cell fate conversions (related to Figure 5)

(A-B) The expression of *Hif1α* was modulated with overexpression or sh-RNA-mediated knockdown via a retrovirus system. Energy metabolism was directly controlled by using small-molecule compounds, oligomycin (1 μM) and 2-DG (5 mM). The Oct4GFP$^+$ colonies were counted on day 15 during reprogramming with mES (A) or 5C medium (B). The numbers of three types of Oct4GFP$^+$ colonies were summarized.

(C) RepSox (1 μM) was used on day 2-7 to inhibit early EMT during reprogramming with 5C medium. TGFβ (TGFβ1/2/3, 1ng/ml each) on day 2-7, LSD1 inhibitor (LSD1i), and sequential introduction of Yamanaka factors (OK+M+S) were used to induce early EMT during reprogramming with mES medium. The numbers of three types of Oct4GFP$^+$ colonies on day 15 were summarized.

Data information: Experiments were independently repeated at least five times (n≥5). Error bars represent standard deviations. Additional statistical information was listed in Dataset EV7.

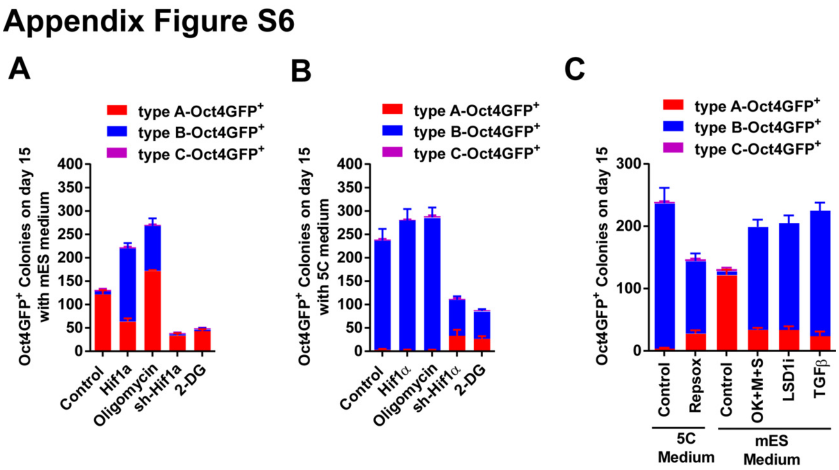

Appendix Figure S7

Comparison with other medium (related to Figure 5)

(A-B) MEFs were reprogrammed with mES, 5C, 5C-naïve (naïve medium was used to replace 5C medium on day 10), iSF1, and iCD1 medium (1:5 passage on day 9) (A). The numbers of three types of Oct4GFP^{+} colonies on day 15 were summarized in (B). The expression of E-cadherin (epithelial marker) and N-cadherin (mesenchymal marker) was determined with qPCR on day 6 during reprogramming (C).

(D-E) Different concentrations of BMP4 (D) and Noggin (E) were used during reprogramming from day 0 to 15 with mES or 5C medium. The numbers of three types of Oct4GFP^{+} colonies on day 15 were summarized.

Data information: Experiments were independently repeated at least five times (n≥5). Error bars represent standard deviations. ***p < 0.001. Additional statistical information was listed in Dataset EV7.

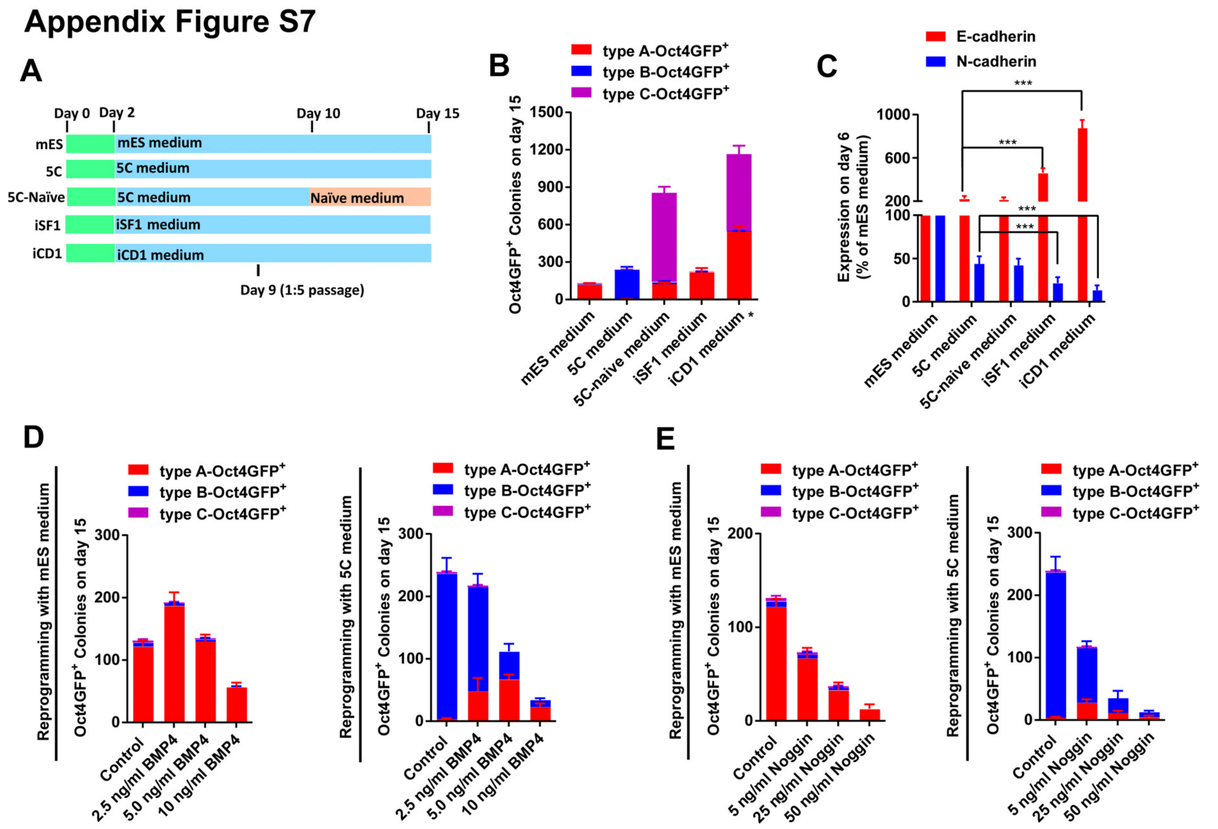

Appendix Figure S8

The positive feedback between EMT and OGS at the late stage (related to Figure 6)

Oct4GFP^{+} and Oct4GFP^{-} cells were separated with FACS on day 15 during reprogramming. 5C-Oct4GFP^{+}, 5C-Oct4GFP^{-}, mES-Oct4GFP^{+}, and mES-Oct4GFP^{-} cells were isolated and analyzed with RNA-Seq. MEFs were used as control and fro normalization.

(A-B) In mES-Oct4GFP^{+} cells, the upregulated epithelial markers (A), downregulated mesenchymal markers (A), upregulated glycolysis markers (B), and downregulated OXPHOS markers (B) were selected from the markers listed in Dataset EV4. The expression of these genes in 5C-Oct4GFP^{+}, 5C-Oct4GFP^{-}, mES-Oct4GFP^{+}, and mES-Oct4GFP^{-} cells was summarized.

(C-D) The averaged expression (Log_2 values) of all epithelial, mesenchymal, glycolysis and OXPHOS markers listed in Dataset EV4 was calculated in 5C-Oct4GFP^{+}, 5C-Oct4GFP^{-}, mES-Oct4GFP^{+}, and mES-Oct4GFP^{-} cells. The resulted values were used to plot the four kinds of cells to show their epithelial-mesenchymal characteristic (C) and glycolysis-OXPHOS characteristic (D).

Data information: Additional statistical information was listed in Dataset EV7.

Appendix Figure S8

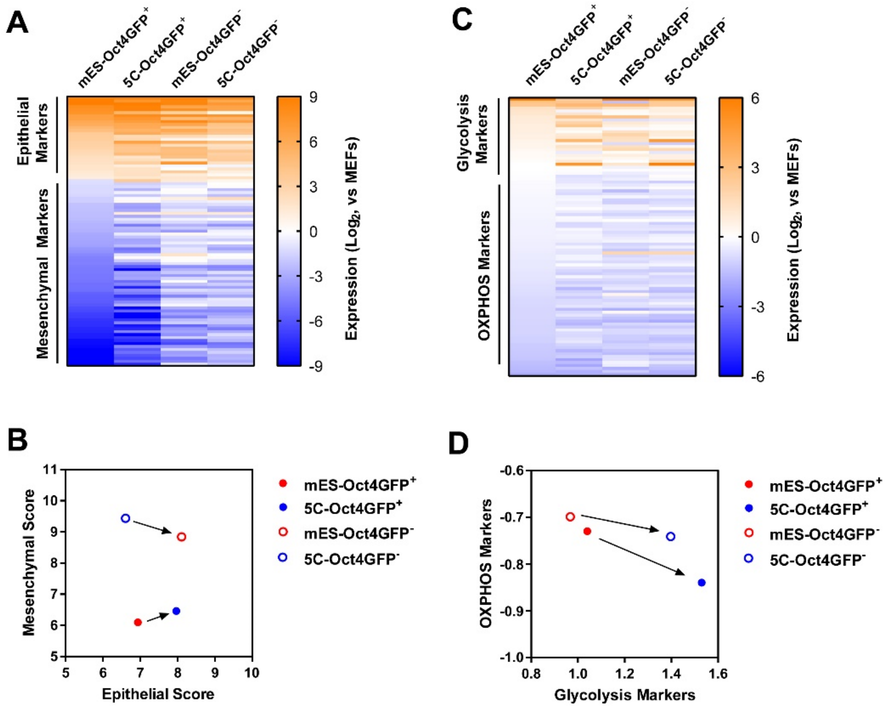

Appendix Figure S9

PSCs in 5C state can be stabilized (related to Figure 6)

5% O_2 and TGFβ (TGFβ1/2/3, 1ng/ml each) were used to culture 5C-Oct4GFP$^+$ colonies for five passages (1:2). Expression of pluripotency markers (A) and percentages of Oct4GFP$^+$ cells (B) were determined with qPCR and FACS, respectively. P0 and P5 suggested no additional passage and addition passage for five times, respectively.

Data information: Experiments were independently repeated at least five times (n≥5). Error bars represent standard deviations. ***p < 0.001. Additional statistical information was listed in Dataset EV7.

Appendix Figure S9

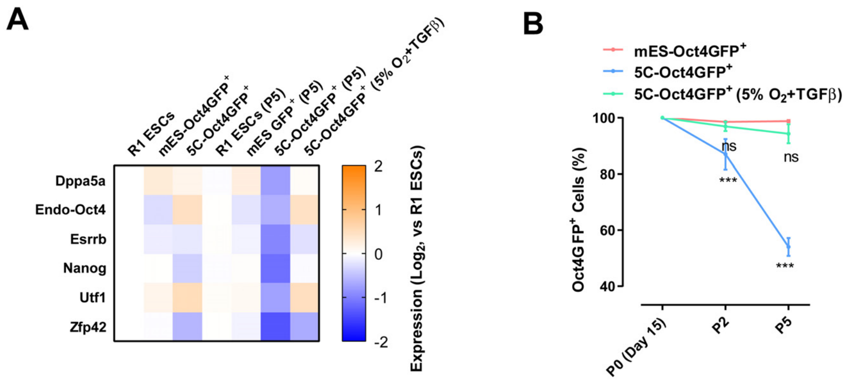

Appendix Figure 10

***Hif2α* is also involved in the cooperation between EMT and OGS (related to Figure 6)**

(A) The expression of *Hif1α* and *Hif2α* was determined on day 6 during reprogramming or in Oct4GFP$^+$ colonies on day 15 with qPCR.

(B) The expression of *Hif1α* and *Hif2α* was modulated in MEFs via a retrovirus system. The expression of mesenchymal markers were determined on day 3 with qPCR.

(C) The expression of *Hif1α* and *Hif2α* was modulated during reprograming with mES medium. The expression of *Bmi1*, *Ctcf*, *Ezh2*, *Kdm2b*, and *Wdr5* was determined on day 6 with qPCR.

Data information: Experiments were independently repeated at least five times (n≥5). Additional statistical information was listed in Dataset EV7.

Appendix Figure S10

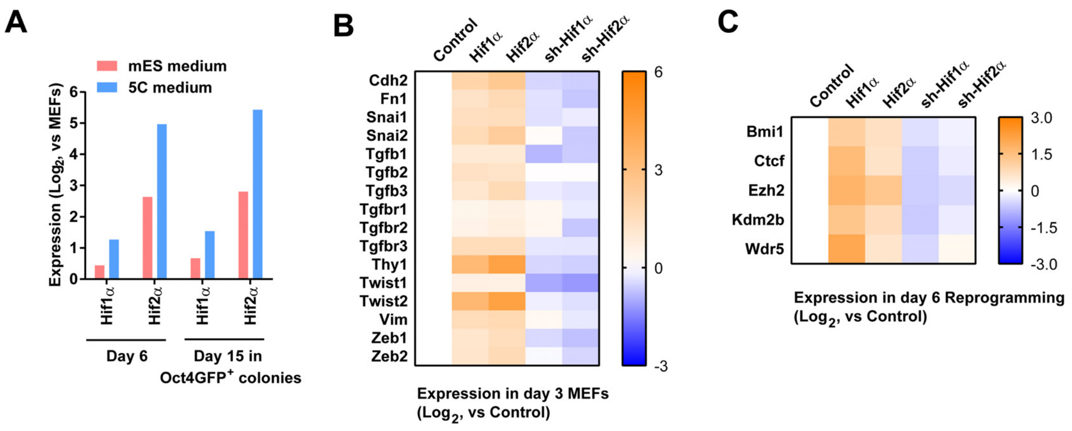

Supplement: Supplementary file 1 — Appendix [file EMBJ-39-e102961-s001.pdf]
