# Supplementary material for: Metabolic switch and epithelial–mesenchymal transition cooperate to regulate pluripotency
Source: EMBO J. 2020 Feb 24;39(8):e102961. doi: 10.15252/embj.2019102961 (PMC7156961; doi:10.15252/embj.2019102961)
Supplement: Supplementary file 2 — Expanded View Figures PDF [file EMBJ-39-e102961-s002.pdf]

Expanded View Figures

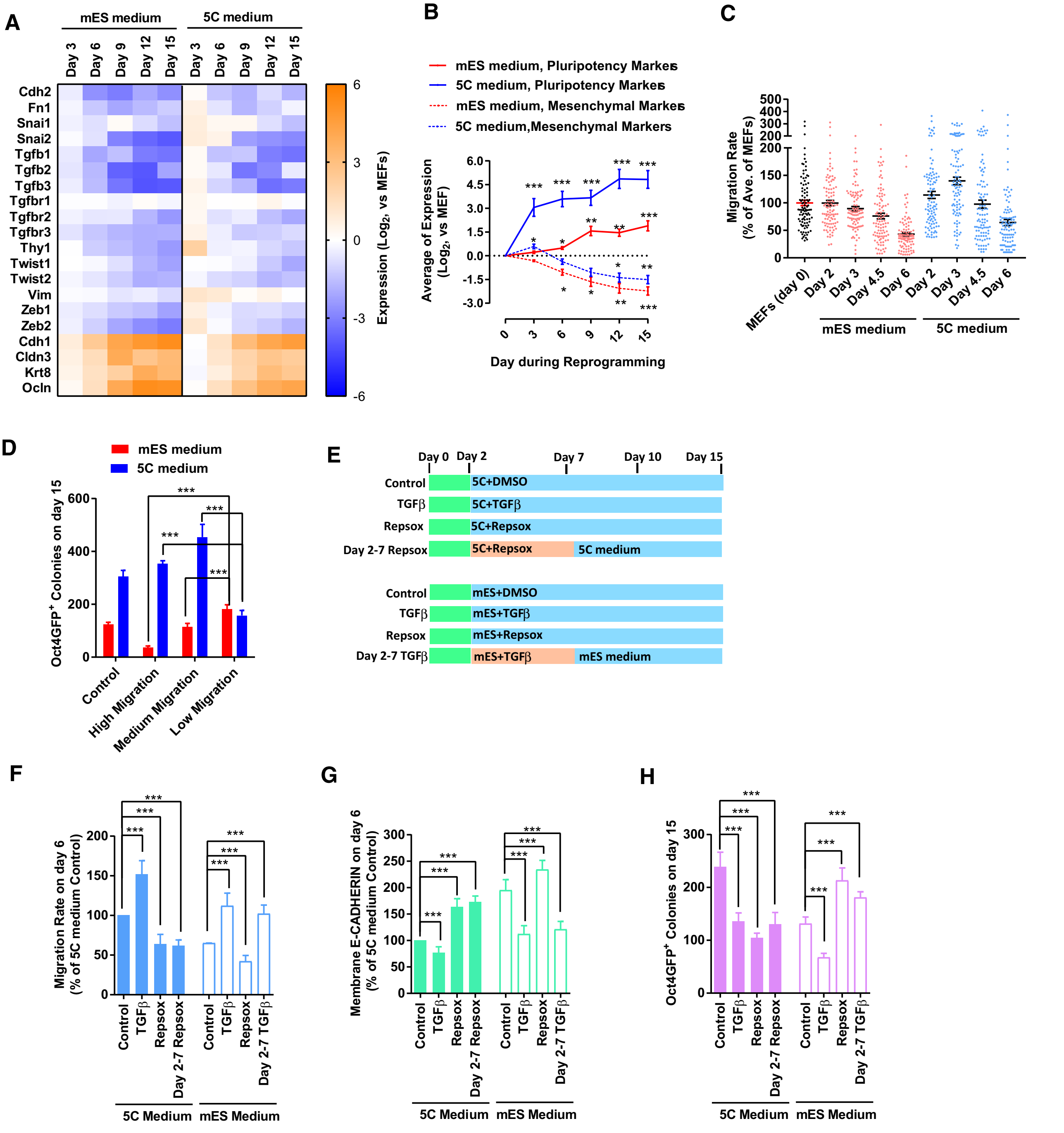

Figure EV1.

Figure EV1. 5C medium promotes reprogramming by inducing early EMT (related to Fig 1).

A Expression of several epithelial and mesenchymal markers was determined with qPCR at different time points during reprogramming with 5C or mES medium.

B The average expression of pluripotency markers and mesenchymal markers was calculated and plotted at different time points during reprogramming based on Figs 1C and EV1A.

C Cell migration was determined at the early stage (days 0–6) during reprogramming with live-cell imaging. Live-cell imaging recorded the actual distance traveled by each individual cell within 1 h.

D Cells were separated into three groups (high, one-third of cells with the highest migration; low, one-third of cells with the lowest migration; and medium, the other one-third of cells) based on their migration abilities on day 3 during reprogramming. Reprogramming of these cells was traced via live-cell imaging. The number of Oct4GFP$^+$ colonies on day 15 converted from different groups of cells was summarized. "Control" was the average of Oct4GFP$^+$ colonies generated from these three groups of cells.

E–H TGFβ (TGFβ1/2/3, 1 ng/ml each) and RepSox (1 μM) were used during reprogramming from days 2–7 or during the whole process (E). The cell migration (F) and E-CADHERIN expression (G) were determined on day 6 with live-cell imaging and FACS, respectively. The number of Oct4GFP$^+$ colonies (H) was determined on day 15.

Data information: Experiments were independently repeated at least five times ($n \geq 5$). Error bars represent standard deviations. *$P < 0.05$, **$P < 0.01$, ***$P < 0.001$. Additional statistical information is listed in Dataset EV7.

© 2020 The Authors

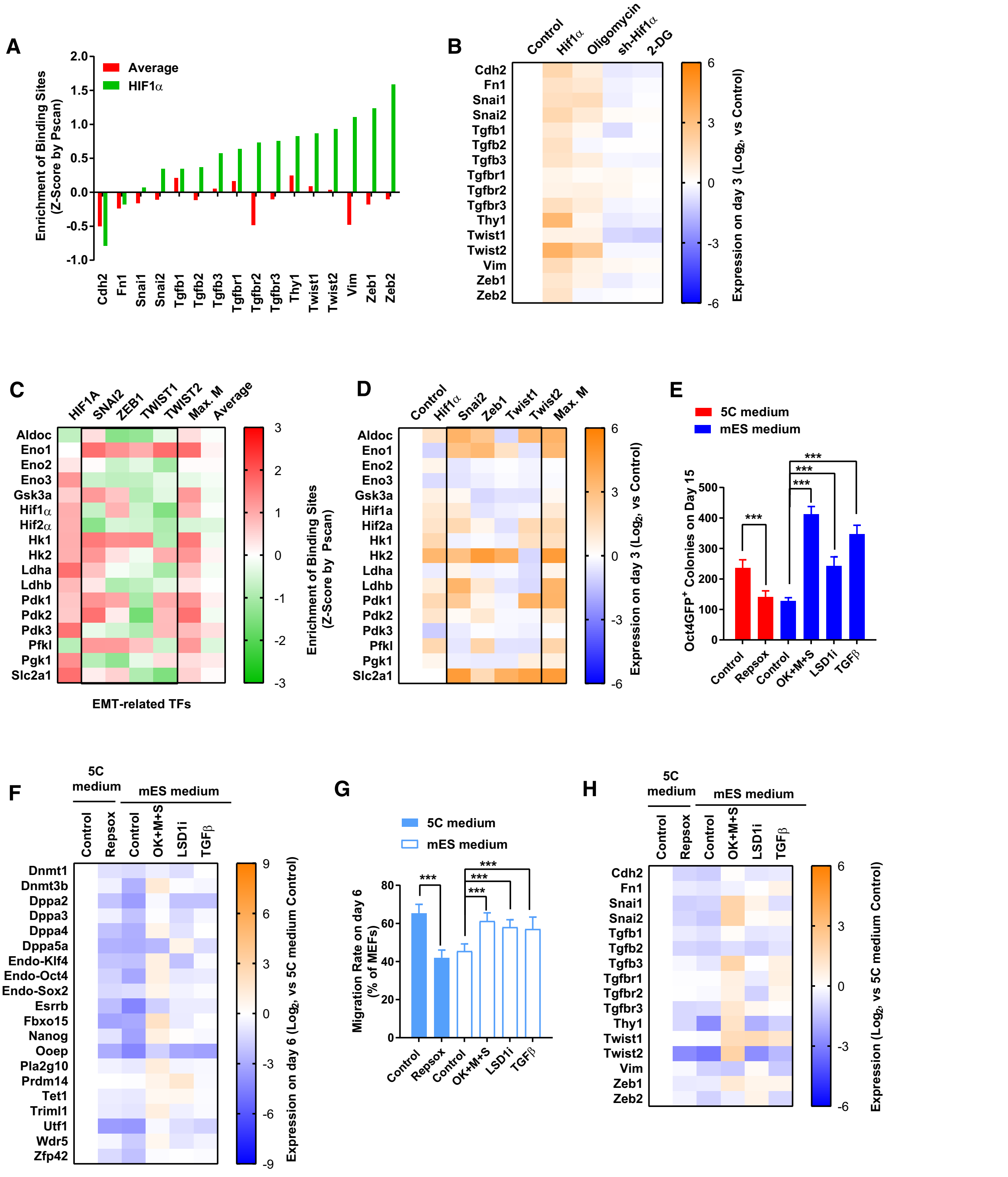
A
Average
HIF1α
Enrichment of Binding Sites (Z-Score by Pscan)
Cdh2 Fn1 Snai1 Snai2 Tgfb1 Tgfb2 Tgfb3 Tgfbr1 Tgfbr2 Tgfbr3 Thy1 Twist1 Twist2 Vim Zeb1 Zeb2
B
Control Hif1α Oligomycin sh-Hif1α 2-DG
Expression on day 3 (Log2, vs Control)
C
HIF1A SNAI2 ZEB1 TWIST1 TWIST2 Max. M Average
Aldoc Eno1 Eno2 Eno3 Gsk3a Hif1α Hif2α Hk1 Hk2 Ldha Ldhb Pdk1 Pdk2 Pdk3 Pfkl Pgk1 Slc2a1
EMT-related TFs
Enrichment of Binding Sites (Z-Score by Pscan)
D
Control Hif1α Snai2 Zeb1 Twist1 Twist2 Max. M
Expression on day 3 (Log2, vs Control)
E
5C medium
mES medium
Oct4GFP+ Colonies on Day 15
Control Repsox Control OK+M+S LSD1i TGFβ
F
5C medium
mES medium
Control Repsox Control OK+M+S LSD1i TGFβ
Dnmt1 Dnmt3b Dppa2 Dppa3 Dppa4 Dppa5a Endo-Klf4 Endo-Oct4 Endo-Sox2 Esrrb Fbxo15 Nanog Ooep Pla2g10 Prdm14 Tet1 Triml1 Utf1 Wdr5 Zfp42
Expression on day 6 (Log2, vs 5C medium Control)
G
5C medium
mES medium
Migration Rate on day 6 (% of MEFs)
H
5C medium
mES medium
Expression (Log2, vs 5C medium Control)

Figure EV2.

Figure EV2. OGS and EMT induce each other via transcriptional activation (related to Fig 3).

A The enrichment of binding sites of HIF1α was determined on the promoters of several key mesenchymal markers with Pscan software. "Average" represented the average *Z*-score of all transcriptional factors tested in with Pscan software (Dataset EV2).

B Expression of *Hif1α* was modulated with overexpression or sh-RNA-mediated knockdown via a retrovirus system in MEFs. Oligomycin (1 μM) or 2-DG (5 mM) was used to treat MEFs. The expression of mesenchymal markers was determined with qPCR on day 3.

C The enrichment of binding sites of indicated transcriptional factors was determined on the promoters of several key glycolysis markers. "Max. M" suggested the maximum *Z*-score generated with SNAI2, TWIST1/2, and ZEB1 binding sites on one indicated promoter. "Average" suggested the average *Z*-score of all transcriptional factors tested in the Pscan software on one indicated promoter.

D Expression of *Hif1α* and key mesenchymal transcriptional factors, including *Snai1/2*, *Twist1/2*, and *Zeb1/2*, was modulated in MEFs. The expression of several key glycolysis markers was determined with qPCR on day 3. "Max. M" indicated the largest upregulation induced by *Snai1/2*, *Twist1/2*, and *Zeb1/2* on one particular gene.

E–H RepSox (1 μM) was used on days 2–7 to inhibit early EMT during reprogramming with 5C medium. TGFβ (TGFβ1/2/3, 1 ng/ml each) on days 2–7, LSD1i (SP2509, 10 nM), and sequential introduction of Yamanaka factors (OK + M + S) were used to induce early EMT during reprogramming with mES medium. Except for OK + M + S group, the four Yamanaka factors were delivered simultaneously. The number of $Oct4GFP^{+}$ colonies was determined on day 15 (E). The expression of pluripotency markers was determined on day 6 with qPCR (F). Cell migration was determined with live-cell imaging on day 6 (G). The expression of mesenchymal markers was determined on day 6 with qPCR (H).

Data information: Experiments were independently repeated at least five times ($n \geq 5$). Error bars represent standard deviations. ***$P < 0.001$. Additional statistical information is listed in Dataset EV7.

 © 2020 The Authors

A

	Naïve	5C	Primed	Formative	References	EpiLCs	References
Morphology	Domed	Flat	Flat	Flat	Kallan et. al., 2017	Flat	Hayashi et. al., 2011
Naïve Markers	High	High	Low	Low	Datasets	Low	Datasets
Primed Markers	Low	Medium	High	Low	Datasets	Low	Datasets
XX State	XaXa	XaXa	XaXi	Partial	Smith, 2017	XaXa	Takahashi et. al., 2018
Chimera	Yes	No	No	Yes	Smith, 2017	Yes	Du et. el., 2018
Germ Cell Competence	No	No	No	Yes	Smith, 2017	Yes	Hayashi et. al., 2011
Epithelial Markers	High	High	High	High	Datasets	High	Datasets
Mesenchymal Markers	Low	Medium	High	Low	Datasets	Low	Datasets
Glycolysis Markers	Low	High	Medium	Medium	Datasets	Medium	Datasets
OXPHOS Markers	High	Medium	Low	Medium	Datasets	Medium	Datasets

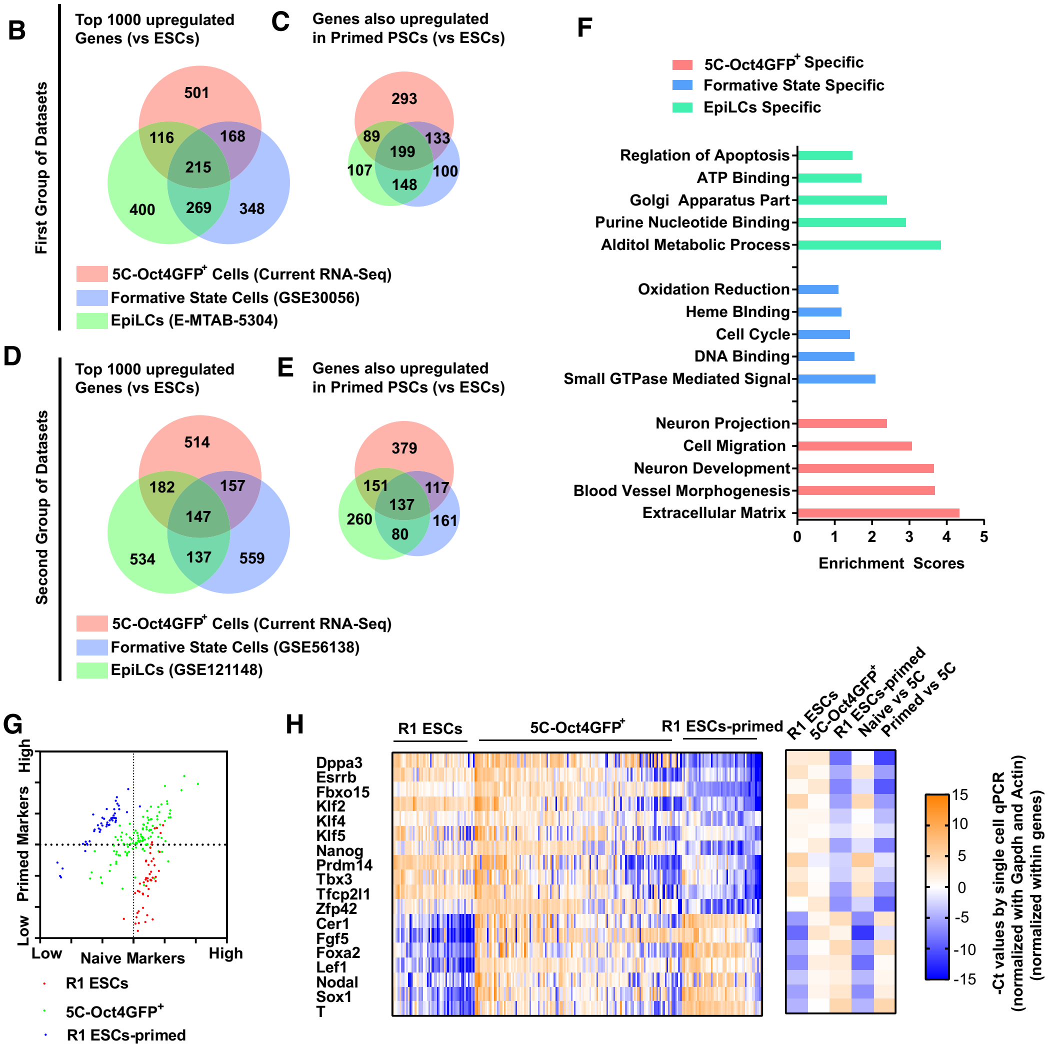

Figure EV3. 5C state is a new pluripotency state (related to Fig 5).

A Summary of characteristics of PSCs in 5C state, formative state, and EpiLCs state.

B, C Top 1,000 upregulated genes (compared to ESCs) were selected from the current RNA-Seq and previously reported datasets (Data ref: Hayashi *et al*, 2011; Data ref: Bertone, 2014) for the three states. The overlapping was determined (B). In addition, the genes which were upregulated in primed PSCs (compared to ESCs, based on the current RNA-Seq) were further selected and listed in (C).

D, E Two different datasets were used to repeat the studies in (B and C) (Data ref: Buecker, 2014; Data ref: Kalkan *et al*, 2019).

F The genes which were only upregulated in one of the three states were used for GO analysis.

G, H Single-cell qPCR was performed with 5C-Oct4GFP$^+$ cells (120 cells), R1 ESCs (48 cells), and R1 ESCs-primed (cells). *Dppa3*, *Esrrb*, *Fbxo15*, *Klf2/4/5*, *Nanog*, *Prdm14*, *Tbx3*, *Tfcp2l1*, and *Zfp42* were used as naive markers, while *Cer1*, *Fgf5*, *Foxa2*, *Lef1*, *Nodal*, *Sox1*, and *T* were used as primed markers. The average expression of naïve and primed markers was calculated for each cell and was used to plot the cells in (G). Heatmap summarized results (H, left), and the average expression of each gene in 5C-Oct4GFP$^+$ cells (5C), R1 ESCs (naïve), and R1 ESCs-primed (primed) was provided and compared (H, right).

Data information: Statistical information is listed in Dataset EV7.

© 2020 The Authors

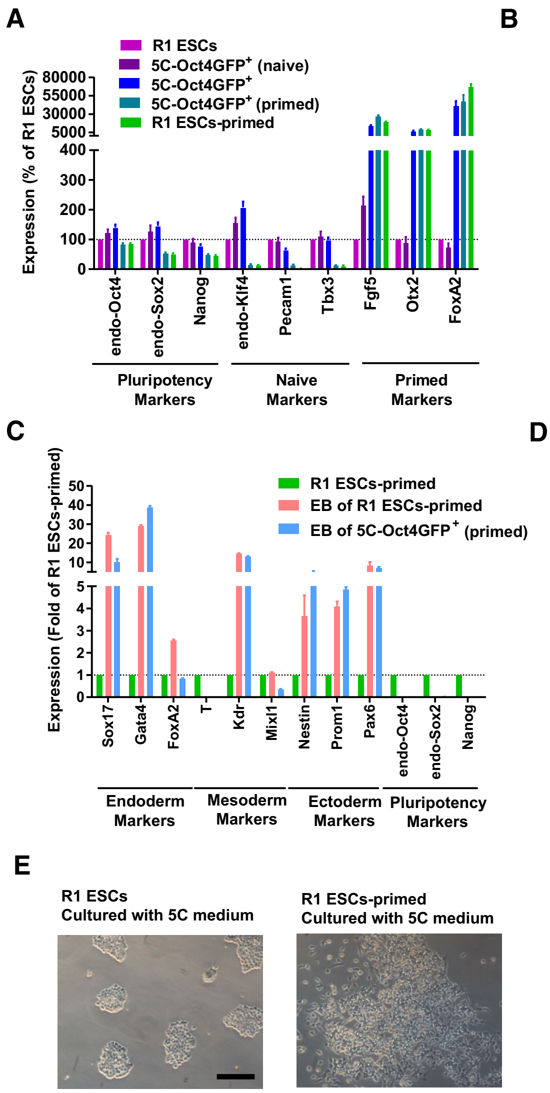

Figure EV4. PSCs in 5C state can be converted to naïve or primed state (related to Fig 5).

A The isolated 5C-Oct4GFP+ cells were cultured in naïve or primed medium for 3 days. The expression of markers of different pluripotent states was determined with qPCR. R1 ESCs and R1 ESCs-primed were used as controls.

B, C The isolated 5C-Oct4GFP+ cells were cultured in naïve and primed medium, and the obtained cells were used for EB differentiation. The expression of markers of endoderm, mesoderm, ectoderm, and pluripotency was determined together with R1 ESCs and R1 ESCs-primed.

D, E R1 ESCs and R1 ESCs-primed were cultured with 5C medium for 3 days, the expression of markers of different pluripotent states was provided in (D), and the morphologies were presented in (E, Scale bar, 100 μM).

Data information: Experiments were independently repeated at least five times ($n \geq 5$). Error bars represent standard deviations. Statistical information is listed in Dataset EV7.

 © 2020 The Authors

Figure EV5. 5C medium facilitates the generation of naïve and primed PSCs (related to Fig 5).

A–C MEFs were reprogrammed with 5C, mES, naïve, or primed medium. In three additional groups, mES, naïve, or primed medium was used to replace 5C medium from day 10 (A). The morphology and OCT4 fluorescence of seven types of colonies were provided in (B, Scale bar, 100 μM). These seven types of colonies were isolated on day 15 during reprogramming with different protocols. The expression of markers of different pluripotent states was determined with qPCR in these colonies (C).

D–H The percentages of Oct4GFP$^+$ cells (D) and the numbers of seven types of colonies (E and F) were summarized on day 15. The expression of *Xist* (G) and the abilities to form chimeras (H) were determined in type C-Oct4GFP$^+$ colonies in 5C-naïve group, type B-Oct4GFP$^+$ colonies in 5C group, and type E-Oct4GFP$^-$ colonies in 5C-primed group.

I–L MEFs were reprogrammed with mES medium. Naïve or primed medium was used to replace mES medium from day 10 (I). The percentages of Oct4GFP$^+$ cells (J) and the numbers of seven types of colonies (K and L) were summarized on day 15.

Data information: Experiments were independently repeated at least five times ($n \geq 5$). Error bars represent standard deviations. ***$P < 0.001$. Additional statistical information is listed in Dataset EV7.

© 2020 The Authors

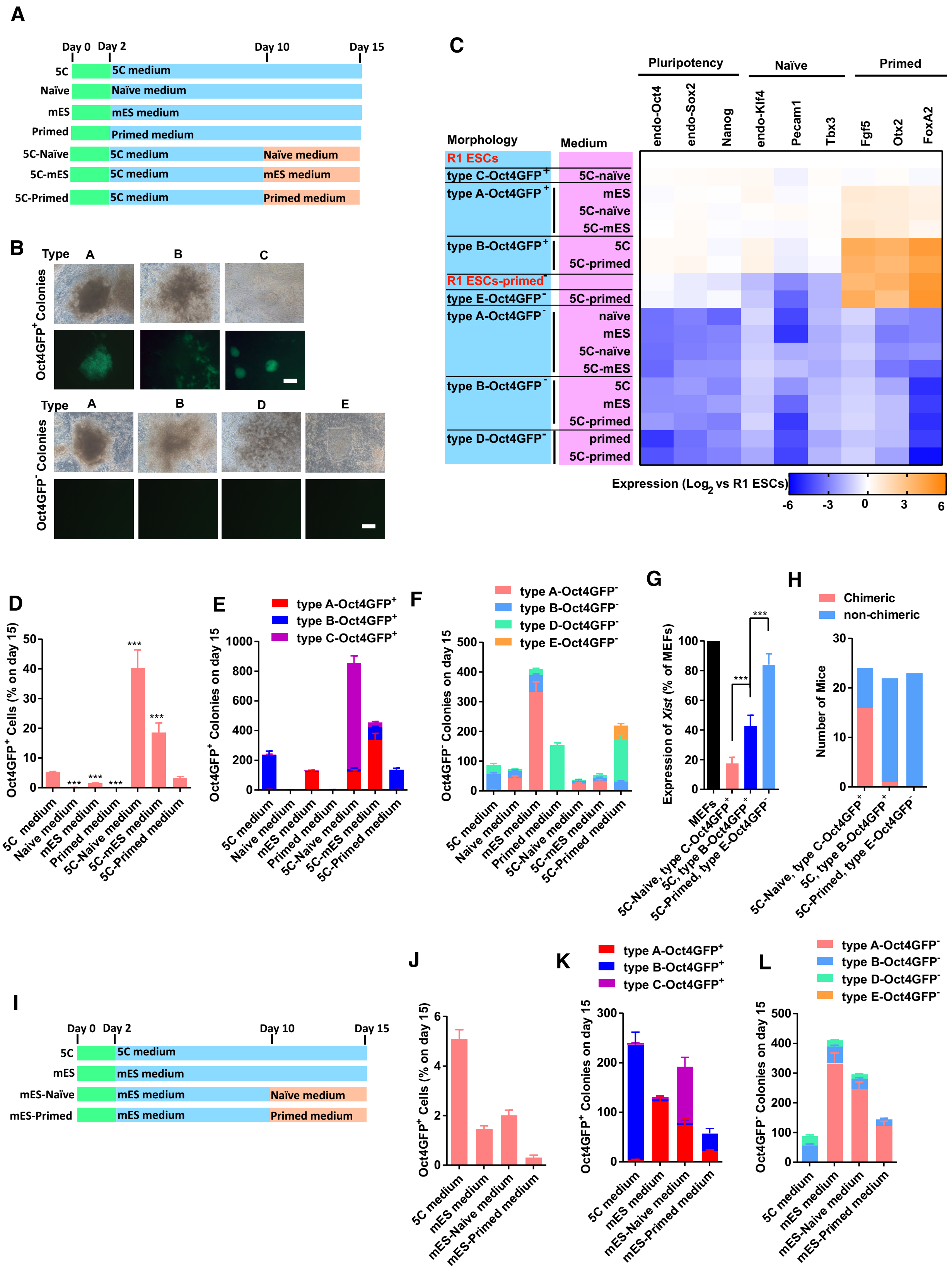

Figure EV5.

© 2020 The Authors
